# Nanomaterials as an alternative to increase plant resistance to abiotic stresses

**DOI:** 10.3389/fpls.2022.1023636

**Published:** 2022-10-11

**Authors:** Humberto Aguirre-Becerra, Ana Angélica Feregrino-Pérez, Karen Esquivel, Claudia Elena Perez-Garcia, Ma. Cristina Vazquez-Hernandez, Aurora Mariana-Alvarado

**Affiliations:** ^1^ Cuerpo Académico de Bioingeniería Básica y Aplicada, Facultad de Ingeniería - Campus Amazcala, Universidad Autónoma de Querétaro, Querétaro, Mexico; ^2^ Facultad de Ingeniería, Universidad Autónoma de Querétaro, Querétaro, Mexico; ^3^ Facultad de Química, Universidad Autónoma de Querétaro, Querétaro, Mexico; ^4^ Cuerpo Académico de Innovación en Bioprocesos Sustentables, Depto. De Ingenierías, Tecnológico Nacional de México en Roque, Guanajuato, Mexico; ^5^ Facultad de Ciencias Naturales, Universidad Autónoma de Querétaro, Querétaro, Mexico

**Keywords:** nanoparticles, nanomaterials, abiotic stress, plant eustress, secondary metabolites, elicitor

## Abstract

The efficient use of natural resources without negative repercussions to the environment has encouraged the incursion of nanotechnology to provide viable alternatives in diverse areas, including crop management. Agriculture faces challenges due to the combination of different abiotic stresses where nanotechnology can contribute with promising applications. In this context, several studies report that the application of nanoparticles and nanomaterials positively affects crop productivity through different strategies such as green synthesis of nanoparticles, plant targeted protection through the application of nanoherbicides and nanofungicides, precise and constant supply of nutrients through nanofertilizers, and tolerance to abiotic stress (e.g., low or high temperatures, drought, salinity, low or high light intensities, UV-B, metals in soil) by several mechanisms such as activation of the antioxidant enzyme system that alleviates oxidative stress. Thus, the present review focuses on the benefits of NPs against these type of stress and their possible action mechanisms derived from the interaction between nanoparticles and plants, and their potential application for improving agricultural practices.

## Introduction

**Figure 1:**
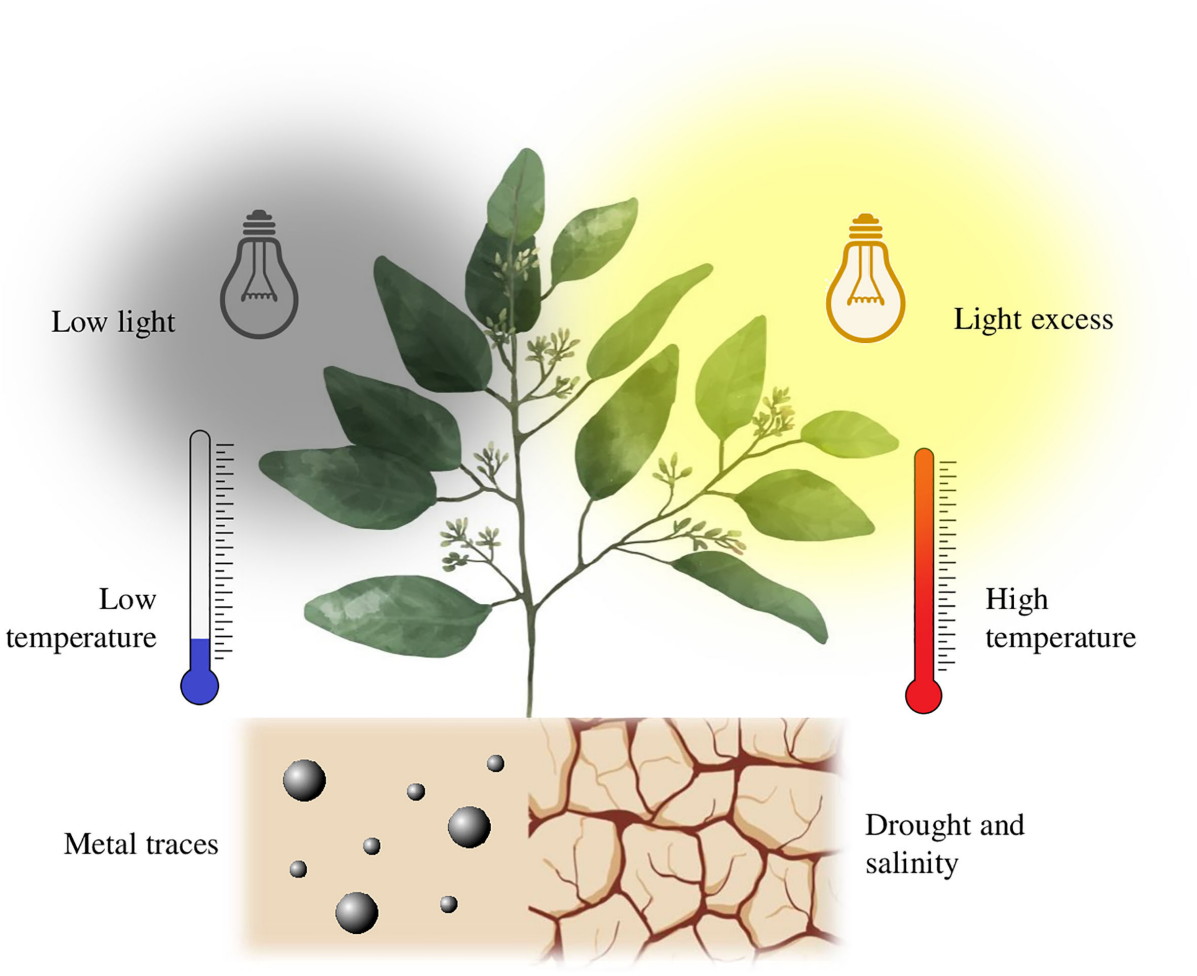
Drought and salinity, temperature, soil contamination and light stress on plants.

Climate change, water deficit, low nutrient use efficiency, crop pests, and decreasing availability of land for crop development, combined with the increasing demand for food due to a growing world population, require the sustainable development of agriculture (Seleiman et al., 2021; Ayub et al., 2022; Gil-Díaz et al., 2022). The efficient use of natural resources without negative repercussions to the environment has encouraged the incursion of new technologies, such as nanotechnology to provide viable alternatives in diverse areas, including agriculture. According to the definition, first coined in 1974 at the Tokyo University of Science, nanotechnology is simply the manipulation of matter at the nanometer scale, and nanoparticles (NPs) are defined as molecules manufactured or synthesized at this scale (Khan and Rizvi, 2014; Seleiman et al., 2020; Badawy et al., 2021; Yu et al., 2021). Nanotechnology involves the utilization of NPs within a size range of 1 to 100 nm with exceptional characteristics such as a high surface/volume ratio and various physicochemical and biochemical properties, which depends on their high surface energy (Dahoumane et al., 2017; Sukhanova et al., 2018) (Ijaz et al., 2020). provides detailed information on the classification of NPs and NMs, where a general classification is into organic (e.g., ferritin, micelles, dendrimers, and liposomes) and inorganic. However, according to their size, shape, structure, and synthesis, they can be more specifically classified into metals (e.g., aluminum, gold, iron, lead, silver, cobalt, zinc, cadmium, and copper), metal oxides (e.g., zinc oxide, silicon dioxide, iron oxide, aluminum oxide, cerium oxide, titanium oxide, and magnetite), ceramics, bio-NPs (e.g., magnetosomes, exosomes, lipoproteins, and viruses), and carbon-based NMs (fullerenes, carbon nanotubes (CNT), graphene, carbon black and carbon nanofibers) (Ijaz et al., 2020; Khan and Hossain, 2022). Novel agricultural applications of nanotechnology in agriculture has focused on the research for the activation of the secondary metabolism in plants, a mechanism of adaptation and evolution as a defense to harsh environmental conditions (biotic and abiotic) (Aguirre-Becerra et al., 2021). The effect of NPs in plant protection against abiotic stress has been documented in several studies. Several applications of NPs and NMs have been proposed and studied to achieve this goal. For example, producing nanocomposites containing fertilizers, pesticides, and microorganisms is one of the most common applications (Usman et al., 2020). Nanoencapsulation of macronutrients, micronutrients, microorganisms, and organic compounds for nano-biofertilization has demonstrated an interesting synergy between crop and substrate microorganisms (Sambangi et al., 2022). Additionally, green methodologies for synthesizing NPs through extracts of plant, fungi, and algae that minimize environmental impact has demonstrated lower phytotoxicity and less environmental impact than the traditional physicochemical approach (Kaur and Sidhu, 2021). Those features impact the production of plant growth regulators and the activation of the secondary metabolism to produce bioactive compounds that protect plants from abiotic stress. Thus, the present article reviews the recent developments on applications of nanotechnology in agriculture, focusing on the benefits of NPs and NMs against drought and salinity, temperature, soil contamination and light stress (**Figure 1**) and their possible action mechanisms without ignoring the potentially toxic effects derived from the interaction between NPs-Plant-Substrate.

## Agricultural potential and plant effect of NPs

The characteristics of NPs and NMs are adequate and promising for improving plant traits and their implementation in agricultural practices. Dimensions smaller than 100 nm, high surface-volume ratio, slow and sustained release potential, diverse nature (i.e., organic, inorganic, and hybrid), shapes, textures, and surface charge diversity, specific interaction, and stability, are useful characteristics of NPs that generate a high potential for their implementation in agricultural practices (Farokhzad and Langer, 2009; Paramo et al., 2020) (Ali et al., 2021; Ali et al., 2021). indicate that the aforementioned characteristics of NPs increase their permeability, promoting cell membrane penetration, and therefore, facilitating their entrance through the leaves and roots of plants (Liscano et al., 2000). Additionally, hollow formations can protect against degradation processes and increase flexibility and elasticity in non-rigid structures, although they can also provide rigidity and compactness to solid structures. The diverse behavior of NPs also makes them viable for developing nanopesticides, nanoherbicides, and nanosensors for plant, soil, and pest monitoring in an intelligently controlled environment, a trend in agriculture 4.0 (Kah et al., 2019; Rastogi et al., 2019; Shawon et al., 2020). However, some of the mentioned physical and chemical characteristics are also related to phytotoxicity, potential environmental toxicity, and human health hazards (Paramo et al., 2020; Ayub et al., 2022), which involves mandatory experimentation to find the appropriate doses, exposure times, and types of NP types for every crop.

The incursion of nanotechnology in agriculture is relatively recent, where the production of nanocomposites containing fertilizers, pesticides, and microorganisms is one of the most important applications (Usman et al., 2020; Elshayb et al., 2022). For instance, nano-biosensors are playing an important role in revolutionizing farming through the development of diagnostic tools and techniques for identifying heavy metal (HM) ions, pollutants, microbial load, and pathogens, along with fast environmental monitoring. Some are placed on the leaves to measure the hydrogen peroxide, a molecule for plant “communication,” producing physical signals (e.g., electric and light) that can be observed and analyzed (Johnson et al., 2021). Nanotechnology also contributes to remediation strategies for contaminated soils that are important regarding the mobility, fate, and toxicity of soil pollutants that considerably decrease crop yield due to plant stress (Usman et al., 2020). In this regard, NPs and NMs can also provide plant protection from abiotic stress conditions (e.g., drought, salinity, low temperatures, UV rays, and toxic trace metals) by several mechanisms such as activating the antioxidant enzyme system that alleviates oxidative stress.

Nanoencapsulation of nutrients or microorganisms for nanofertilization is becoming popular in agriculture because the material that constitutes the NPs can generate synergy with the crop and the microorganisms present in the substrate, providing plants with compounds that are important for their development (Tan et al., 2017). For example, nanoencapsulated B was foliar applied to sweet potato plants, increasing B, Fe, and Mn concentration in leaves, and NIP aquaporins and Boron transporters expression (Nicolas-Espinosa et al., 2022). Nano-chelated Fe fertilizer on rice increased biological yield by 27%, decreased hollow grain number by 254% and raised protein content and N, P, K, Fe, and Zn concentrations (Fakharzadeh et al., 2020). In the same way, the combination of FeNPs, ZnNPs, and CuNPs with a commercial fertilizer in a tomato crop increased the plant’s height, stem diameter, root length, photosynthetic pigments, leaf minerals, and protein, fiber, Fe, Zn, and K, and antioxidant properties of tomatoes (Rahman et al., 2021). Additionally, the fertilization with NPK with the spraying of VNPs and SeNps in rice increased plant height, chlorophyll index, biological yield, grain yield, harvest index, and agronomic efficiency (Al-Karaawi and Al-Juthery, 2022). Nanoencapsulation of microorganisms or organic compounds is a trend in nano-biofertilizing. For example, growth-promoting rhizobacteria and their metabolites were nanoencapsulated using SiNPs and carbon nanotubes to improve pistachio micropropagation, where the use of Pseudomonas fluorescens VUPF5 and Bacillus subtilis VRU1 nanocapsules significantly enhanced the root length, shoot length, fresh weight, and proliferation (Moradipour et al., 2019). Moreover, a foliar application of nano-chitosan under different phosphorus fertilizer levels on hot pepper increased plant height, the number of branches per plant, total plant dry weight, fruit set, fruit length, fruit diameter, yield, total chlorophyll, total soluble solids, vitamin C, total nitrogen, total phosphorus, total potassium, and capsaicin content (Helaly and EL-Bauome, 2020).

In addition to mitigating environmental stress on crops, one of the trends in this area is the search for green methodologies for the synthesis of NPs that minimize environmental impact. Different techniques to synthesize NPs result in compounds with different shapes, sizes, and biological activities and applications in different areas (Ali et al., 2021; Ali et al., 2021). In recent times, the synthesis of metal and metal oxide NPs using plant, fungi and algae extracts has recently emerged in the field of nanoscience and nanobiotechnology, with numerous advantages over conventional physicochemical approaches (Karthik et al., 2022; Srivastava and Bhargava, 2022a; Srivastava and Bhargava, 2022b). Chemical reduction is the most common procedure to synthetize NPs. Other chemical techniques include electrochemical techniques, solvo-thermal reduction, and photochemical reaction in reverse micelles (Gudikandula and Charya Maringanti, 2016). However, the implementation of chemical reducing agents requires considerable amounts of energy and generates byproducts that are dangerous for the environment and human health (Guan et al., 2022). Some bacteria, fungi and plant extracts can be used to synthesize NPs (**Table 1**), simplifying the production process and decreasing the use of solvents and energy, making this green technology increasingly attractive to both scientists and industry (Panpatte et al., 2016; Park et al., 2016; Dahoumane et al., 2017). These environmentally friendly methodologies are cost-effective, rapid, less arduous and more proficient than the conventional chemical procedures and cost-effective, rapid, less arduous and more proficient than the conventional chemical procedures (Willner et al., 2007; Katiyar et al., 2020). Green synthesis of nanoparticles has provided an eco-friendly alternative in agricultural applications (Kaur and Sidhu, 2021). Innovative pest-resistive, eco-friendly and intelligent nano-pesticides have emerged due to the negative impact of traditional pesticides (Dangi and Verma, 2021). Implementing NPs for nanoformulation and nanoencapsulation methods has proven adequate efficiencies in plant pathogenic organisms (e.g., *Streptomyces sannanesis, Bacillus subtilis, Pseudomonas aeruginosa, Salmonella enterica, Candida albicans*, and *Aspergillus niger) *with* *attenuated phytotoxicity (Keerthana et al., 2021). Nanopesticides offer sustained release kinetics, solubility efficiency, enhanced permeability, and stability (Dangi and Verma, 2021). Additionally, this technology has also been implemented as a nanoscale growth regulator in agriculture, improving the yield of many crops (e.g., tomato, rice, cucumber, lettuce, corn, and wheat) (Pelegrino et al., 2020; Tovar et al., 2020; Wahid et al., 2020; Rahman et al., 2021; Al-Karaawi and Al-Juthery, 2022; Ghani et al., 2022).

**Table 1:** Recent examples of the implementation of bacteria, fungi and plant extracts for the synthesis of NPs and NMs.

Extract origin	NPs produced	Reference
Leaves of Origanum majorana	Ag	(Yassin et al., 2022)
Fruit peel of Punica granatum	Zr	(Chau et al., 2022)
Tea leaves of Camellia sinensis	Mg	(Kumar et al., 2022)
Leaves of Allium fistulosum, Tabernaemontana divaricate and Basella alba	Ag	(Vinodhini et al., 2022)
Leaves of Synadium grantii	ZnO	(Karthik et al., 2022)
Leaves of Atriplex halimus	Pt	(Eltaweil et al., 2022)
Leaves of Thymbra Spicata L.	ZnO	(Gur et al., 2022)
Fruits of Conocarpus Lancifolius	Ag	(Oves et al., 2022)
Berries of Ribes rubrum	Ag	(Rizwana et al., 2022)
Fungus Ganoderma lucidum	Ag and Au	(Nguyen et al., 2022)
Fruit peel of Punica granatum	Zero- valent Fe	(Rashtbari et al., 2022)
Endophytic fungi Aspergillus sp isolated from marine seaweed Dictyota dichotoma	ZnO	(Kumar et al., 2022)
Fungus Aspergillus terreus	Cu	(El-Naggar et al., 2022)
Fungus Talaromyces purpureogenus isolated from Taxus baccata Linn	Ag	(Sharma et al., 2022)
Seaweeds Halymenia porphyriformis and Solieria robusta	Ag	(Khan et al., 2022)
Seaweed Champia parvula	Au	(Viswanathan et al., 2022)
Leaves of Eucalyptus globulus	Ag and Ag-TiO_2_	(Torres-Limiñana et al., 2022)

Crop breeding has focused on two main strategies: natural selection and genetic modification, where the latter consists of the insertion of the desired DNA into the plant genome (Godbey, 2022); however, although this technique is considered a reasonably successful technique, it presents several challenges such as cell toxicity and penetration power of foreign DNA, both at the cell wall level and in the genome of the target cell (Krishna et al., 2018). In this regard, nanotechnology provides new tools to enhance gene modification (Singh et al., 2021). In their review (Sheikh Mohamed and Sakthi Kumar, 2019), indicate that a wide range of NPs can be used for the transport and carriage of DNA, RNA, and proteins into the host genome, ensuring successful and correct transgene incorporation. Additionally (Jat et al., 2020), observed that NPs exerted direct and specific penetration into plant sites and organelles such as protoplasts, chloroplasts, pollens, and leaves under transgenic and non-transgenic techniques (Kwak et al., 2019). reported an effective chloroplast translocation using chitosan-functionalized carbon nanotubes, improving plasmid DNA transfer efficiency in chloroplasts. Although their effectiveness and efficacy have been demonstrated, the use of NPs as carriers of DNA, RNA, and proteins is still under discussion and study since the design, toxicity, and effectiveness depend on the type, size, dosage, exposure time, and crop.

Plant growth and development, germination, and production of primary and secondary metabolites are regulated by compounds of chemical nature that are present endogenously in the plant or are obtained from the metabolism of microorganisms or by laboratory synthesis (Bisht et al., 2018). Phytohormones and growth regulators can act punctually in germination, photosynthesis, fruit filling, antioxidant enzyme system, and biotic and abiotic stress alleviation. Studies have demonstrated that NPs and NMs can induce the production of these compounds, enhancing the biological and biochemical processes of plants. For example (Siegel et al., 2018), observed that AuNPs up-regulated the gene expression related to oxidative and metallic stress response in *Arabidopsis thaliana* but down-regulated the gene expression related to pathogen response and exerted various hormonal stimuli such as ethylene signaling and auxin response for tissue growth (Manickavasagam et al., 2019). showed the hormetic influence of biosynthesized Ag NPs on the expression of plant growth regulator genes of regenerating rice calli, finding that he transcript levels of ethylene, ABA, auxin, cytokinin and gibberellic acid responsive genes decreased by a maximum of 0.3, 0.35, 0.2, 0.3 and 0.4 fold, respectively, after culture in medium with 10 mg L^−1^, but above this concentration the expression of all the genes markedly increased, demonstrating that the dose-effect curve is not always linear. Additionally (Pociecha et al., 2021), indicated that the application of two different AgNPs suspensions differentially affected the phytohormone balance in wheat, where after three weeks, the proportions of cytokinins (29%, 42%) and gibberellins (63%, 55%), respectively, were reversed compared to the control.

**Table 2** shows more examples of the application of NPs and NMs that impacted the production of plant growth regulators. Finally, as phytohormones regulates different plant processes, novel research has proposed the implementation of these compounds to increase the efficiency in the process of NPs biosynthesis. For example (Mohamed et al., 2022), found that the addition of indole acetic acid and kinetin to the cyanobacterium *Cyanothece* sp provoked a maximum conversion (87.29% and 55.16%, respectively) for the production of Ag NPs, and that methyl jasmonate increased the NPs conversion rate to 90.29%, although nearly all the cyanobacterial cultures died at the end. Although the effect of various nanoparticles on plant growth and development has been extensively investigated, there are still many questions about their action. For example, in seed priming (Chandrasekaran et al., 2020) establishes that it needs to be first cleared whether IAA have a role in nanoparticle internalization and transport across tissues in primed seeds, that the regulation of carriers involved in transporting nanoparticles from seed coat to endosperm and then to embryonic tissues has not been characterized, whether *in vivo* seed mineral content have a role during internalization of various mineral based nanoparticles, how seeds recognize nanoparticles as external agents, and a detailed description of crosstalk between reactive oxygen species (ROS) and growth regulators due to NPs addition.

**Table 2:** Stimulation of growth regulators by NPs.

NP	Plant/condition	Effect	Reference
C13 (^13^C)-labelled fullerene (C_60_)	Rice (*Oryza sativa*) seedlings	(↓) Dihydrozeatin riboside (23% and 18%), ZR (23% and 18%), ABA (11.1% and 12.7%), brassinolide (12.9% and 13.1%) and GA 4 (12.9% and 13.1%) for 20 mg L−1 and 100 mg L−1, respectively. (↑) GA 3 by 12% and 7%* * for 20 mg L−1 and 100 mg L−1, respectively. (↑) Methyl jasmonate by 19.4% with 100 mg L−1. (↓) IAA by 13.5% with 100 mg L−1.	(Guo et al., 2021)
Ag	Maize (*Zea mays* L) irrigated with municipal wastewater	Modulation of ABA (34%), IAA (55%), and GA (82%), (↑) proline production (70%).	(Khan and Bano, 2016)
CeO_2_	Stress in hydroponic rice (*Oryza sativa*) caused by low N (LN) and high N (HN). MN: normal N dose	(↓) ABA levels in shoots under LN and HN suggesting stress reduction. (↑) ABA levels up to 88% in MN suggesting plant stress. (↑) IAA in HN (177–205% in roots and 112–168% in shoots) and LN (11–161% in roots and 25–32% in shoots), promoting plant growth and stress tolerance. (↓) IAA in MN, inhibiting plant growth. (↑) GA in LN (150% and 70% in roots and shoots, respectively). (↑) GA by 20% in roots in HN. (↑) ZR (a type of cytokinin) under both LN and HN.	(Wang et al., 2020)
SWCNTs and MWCNTs	Rice (*Oryza sativa* L.) seedlings	(↑) GA about 3-fold compared with the control, with MWCNTs by more than 90%. (↓) ABA by 43% with both SWCNTs and MWCNTs.	(Zhang et al., 2017)
TiO_2_	Wheat (*Triticum aestivum* L.) seedlings under super-elevated and normal CO_2_ conditions	(↓) IAA under elevated CO_2_ as TiO_2_ concentrations increased. JA contents exhibited a dose-response manner under super-elevated CO_2_.	(Jiang et al., 2017)

(↑) Increase of; (↓) Decrease of; ABA, Abscisic acid; IAA, Indole-3-acetic acid; GA, Gibberellic acid; ZR, zeatin riboside; SWCNTs, Single-walled carbon nanotubes; MWCNTs, multi-walled carbon nanotubes.

## NPs for plant protection against abiotic stress

Plants are sessile organisms exposed to constant environmental changes, nutrient competition, pests, diseases, water changes, and variations in solar radiation (Vermeulen et al., 2012; Aguirre-Becerra et al., 2021). To counteract these phenomena, plants have developed defense systems consisting of diverse chemical and physical reactions to synthesize compounds that contribute to the response to these stimuli (Alvarado et al., 2019; Aguirre-Becerra et al., 2021). The medicinal value of these compounds, called secondary metabolites, has called for the need for their enhanced production in plants, mainly through plant cell and organ cultures and by providing precursors and elicitors to alter the environmental conditions causing eustress (Khan et al., 2019; Khan et al., 2019). In this sense, according to the hormesis curve of each plant model, the stress can be divided into distress (bad stress that leads to damage and ultimately plant death) or eustress (good stress that leads to activation of secondary metabolism); therefore, the objective of elicitation will be to cause eustress by searching the adequate doses, exposure times, type of application (foliar or irrigation), and type of elicitor (Aguirre-Becerra et al., 2021).

Agriculture faces challenges due to biotic and abiotic stresses where nanotechnology can contribute with promising applications (Usman et al., 2020). In this context, several studies report that the application of NPs positively affects crop productivity through different strategies such as favorable DNA carriers or carriers that improve genetic traits and seeds, plant targeted protection through the application of nanoherbicides and nanofungicides, precise and constant supply of nutrients through nanofertilizers, and tolerance to abiotic stress (e.g., low or high temperatures, drought, salinity, low or high light intensities, UV-B) (Usman et al., 2020; Predoi et al., 2020; Ali et al., 2021). NPs can also act as gene expression modulators in response to abiotic stress conditions (e.g., such as drought, salinity, low temperatures, UV rays, and HMs), providing plant protection by several mechanisms such as activation of the antioxidant enzyme system that alleviates oxidative stress, intervening in biosynthesis and cellular organization, as well as in electron and energy transport (Vishwakarma et al., 2018; Faraji and Sepehri, 2020; Mittal et al., 2020; Sarraf et al., 2022). Examples of the interaction of NPs with different types of abiotic stresses are presented in **Table 3** and the description of the most revised types of abiotic stress are next described.

**Table 3:** Effect of several NPs on plant abiotic stress.

NP	Abiotic stress	Plant/Condition	Effect	Reference
S NPs	Cu-amended medium	Oilseed rape (*Brassica napus* L.)	(↑) Shoot height, root length, and dry weight of shoot and root by 34.6%, 282%, 41.7% and 37.1%, respectively. (↓) Shoot and root Cu contents and MDA levels in shoots and roots by 37.6%, 35%, 28.4% and 26.8%, respectively. (↑) SOD, POD, CAT, APX, GR and GST enzyme activities in shoots by 10.9% to 37.1% and roots 14.6% to 35.3%. S NPs also positively promoted nutrients accumulation in plant (K, Ca, P, Mg, Mn, Zn and Fe).	(Yuan et al., 2023)
Ag NP: 40 ∼ 120 ppm	Salinity: 30 ∼ 120 mM L^−1^ of NaCl	*Satureja hortensis* L. during germination stage	80 ppm improved germination speed, plant height, and stem length*, and despite the gradual increase in salinity levels, this treatment did not stop germination at high salinity. Better use of water and light for photosynthesis in saline conditions.	(Nejatzadeh, 2021)
SWCNTs (50 ∼ 800 μg mL^−1^)	Drought: mild, moderate, and severe drought for 14 days	*Hyoscyamus niger* seed germination	With the lowest dose (50 g mL^−1^) under low and moderate drought: Accumulation of about 22.4% and 7.8% of moisture inside the seeds. (↑) Root length by 12.5% and 26.2% (↓) Accumulation of H_2_O_2_ by 20.9% and 10.6%. Higher doses presented high toxicity.	(Hatami et al., 2017)
Ti NPs (through chemical and green synthesis)	Arsenic toxicity: 10 mM of As	*Vigna radiata* L.: Seeds were soaked in chemical TiNP (0.25%), As + chemical TiNP; and green TiNP (0.1%), and As + green TiNP, for 2 hr.	(↑) Radicle length (39.7% and 62.1%), fresh mass (67.6% and 82.3%), and dry mass (66% and 71.6%) (↓) ROS. O_2_ by 20% and 29%, and H_2_O_2_ by 35% and 43%. (↓) MD by 39% and 44%, suggesting membrane protection. (↑) Protein content by 34% and 59%. (↑) SOD and CAT, approximately 2-fold. All the percentages are for chemical TiNPs + As and green TiNPs + As compared with As treatment, respectively.	(Katiyar et al., 2020)
Ag NPs: 25 ∼ 100 mg L^-1^	High temperature: increased gradually as a step function and maintained between 35–40°C for set time duration (3 h/day).	Wheat (*Triticum aestivum* L.) at three leaf stage	Best results with 50 mg L^-1^: (↑) Relative water content (12.2%), membrane stability index (26.5%), chlorophyll a (10%) and b (16.4%), and total chlorophyll content (19%)*. (↓) Proline (4%) and sugar level (5.8%)*. This compounds are involved in stress tolerance through osmotic adjustment and ROS detoxification (Mishra et al., 2016; Ahmed et al., 2016) (↑) Protein contents (2.6%) and glutathione level (2.3%)*, which protects from membrane degradation and proteolysis process. (↑) TPC (2.4%), TFC (2.5%) and total ascorbates (2.5%)*. Maximum activities of SOD (1.3%), POX (1.5%), CAT (1.8%), APX (1.2%) and GPX (1.4%) were observed*	(Iqbal et al., 2019)
CeO_2_ NPs (foliar)	Salinity (folia application of 200 mM NaCl solution)	Cotton (Gossypium) variety Xinluzao 74	(↑) Chlorophyll content (68%), biomass (38%), and photosynthetic performance such as carbon assimilation rate (144%). (↓) MDA (44%) and ROS level such as H2O2 (79%). (↑) Cytosolic K+ (84%) and leaf K+ (84%). (↓) Cytosolic Na+ (77%) and leaf Na+ (63%)	(Liu et al., 2021)
Fe NPs (Fe_3_O_4_, Fe(II, III) oxide): 5 ∼ 20 mg/kg (foliar and soil)	Contaminated soil with Cd and Fe. Total and available Cd and Fe concentrations (mg/kg) in the soil were 7.38, 0.93, 198.4, and 52.04, respectively	Wheat (*Triticum aestivum* L.)	(↑) Shoot length by 10% ∼ 43% and 21% ∼ 55%^■^ (↑) Spike length by 54% and 60% with 20 mg kg^-1■^ (↑) Shoot dry weight by 11%^■^ 52% and 27% ∼ 72%^■^ (↑) Root dry weight by 50% and 59% with 20 mg kg^-1■^ (↑) Spike and grain dry weights by 90%, and 97% with foliar treatment, and 69% and 74% in soil amendment at 20 mg L^-1^*. (↑) SOD activity by 46% and 40% at 20 mg kg^-1■^ (↑) POD activity by 22% ∼ 54% in leaves at soil application*. ^■^ with soil and foliar application, respectively*.	(Hussain et al., 2019)
Ag: 300 ppm	Salinity: 100 mM of NaCl	Wheat (*Triticum aestivum* L.)	(↑) Seed germination, germination rate, and germination index during the emerging stage. (↓) H_2_O_2_ content by 56% and Thiobarbituric Acid Reactive Substances content by 38%. (↑) SOD, APX, GR, and GPX activity by 44%, 82%, 89%, and 20%, respectively. (↓) Root Na^+^ and Cl^−^ content up to 70% and 73%, respectively, and leaf Na^+^ and Cl^−^ content up to 80% and 83%, respectively. (↑) NR and NiR activities, and content of N by about 60%, 109%, and 98%, respectively (↑) SA by 11.93% (↓) SSC by 15.72% and ABA by about 46% All these results are the comparison of AgNP + NaCl treatment with their respective NaCl treated plants.	(Wahid et al., 2020)
CeO_2_ NPs (transported into chloroplasts)	Excess light (2000 μmol m^-2^ s^−1^, 1.5 h), heat (35°C, 2.5 h), and dark chilling (4°C, 5 days)	*Arabidopsis thaliana*	(↑) 19% in quantum yield of photosystem II, 67% in carbon assimilation rates, and 61% in Rubisco carboxylation rates with a low Ce3^+^/Ce4^+^ ratio (35.0%).	(Wu et al., 2017)
ZnO NPs (seeds soaked in 500 mg L^-1^ solution for 24 h)	Co Stress (CoCl_2_ at a concentration of 300 µM)	Maize (*Zea mays*) seedlings	(↑) Plant growth and fresh biomass accumulation (↓) Co accumulation in root (15%) and shoot (18%) *via* (↑) Plant Zn content by 52% and 73% and general nutrient content in root and shoot. (↓) Co-induced reduction in chlorophyll, SPAD values and photochemical efficiency (↓) ROS accumulation and MDA content in shoot tissues. (↑) APX, CAT and SOD by 15%, 16% and 56%, respectively. All compared with the only Co treated plants. (↓) Damages of stomatal aperture and shrinkage in their guard cells.	(Salam et al., 2022)

(↑) Increase of; (↓) Decrease of; * compared with the control treatment; SWCNTs, single-walled carbon nanotubes; TPC, total phenolic content; TFC, total flavonoid content; MDA, malondialdehyde.

### Drought and salinity stress

The incidence and extent of drought are predicted to increase, creating considerable pressure on global agricultural yields (Seleiman et al., 2021). This type of abiotic stress has the strongest effect on soil biota and plants, generating osmotic stress and reducing nutrient access to plant roots (Zia et al., 2021). Furthermore, soil salinity is the most common abiotic stress with a significant impact on crop production and development, causing massive economic damage in 1,125 million hectares, with 1.5 million hectares of arable land lost each year due to salinization and sodification (Abou-Zeid and Ismail, 2018; Taha R et al., 2020; Alabdallah and Hasan, 2021; Alharbi et al., 2021). Salt stress disrupts the plasma membrane integrity, reduces photosynthetic efficiency, decreases stomata aperture and accessibility of antioxidant enzymes, generating excessive amount of ROS, resulting in cellular oxidative-burst and affecting proteins, DNA, and lipids (Muchate et al., 2016; Tanveer and Ahmed, 2020; Seleiman et al., 2020). Ionic stress is caused by a high accumulation of sodium (Na^+^) and chloride (Cl^−^) ions in plants, leading to disturbance in distribution, uptake, and availability of macro and microelements, and integrity and selectivity of cellular membranes (Thor, 2019). To handle this stress, plants have adaptation mechanisms such as salt ion exclusion, changes in membrane-permeability, synthesis and accumulation of compatible metabolites or osmolytes such as proline, promoting ionic homeostasis, and hormonal regulation such as ABA (Banik and Bhattacharjee, 2020; Van Zelm et al., 2020) that contributes in conserving cellular water by initiating a cascade signaling pathway that leads to efflux of calcium ions (Ca^2+^), NO^3−^ ions, and potassium ions (K^+^) which results in stomatal closure (Medeiros et al., 2019). Positive effects of NPs on plants under drought and salinity stress have been demonstrated by changing the phytohormone concentrations (An et al., 2020), gene expression (Linh et al., 2020), secondary metabolites production (Etesami et al., 2021), increasing nutrient concentration and availability (Mahmoud et al., 2020), decreasing plasma membrane damage and chlorophyll degradation (Karimi et al., 2020), enhancing K^+^ uptake, K^+^/Na^+^ ratio (Abdoli et al., 2020), and activating the antioxidant enzyme activity (Faizan et al., 2018; Moradbeygi et al., 2020; Alabdallah and Alzahrani, 2020). In this way, NPs mitigate drought-induced ROS through the aggregation of osmolytes, which results in enhanced osmotic adaptation and crop water balance (Alabdallah et al., 2021). Furthermore, NPs increase the photosynthetic activity, upregulate aquaporins, modify the intracellular water metabolism, accumulates compatible solutes, maintain intracellular ion homeostasis, increase stomatal density, and reduce water loss from leaves through stomatal closure due to fostered ABA accumulation (Attaran Dowom et al., 2022; Kandhol et al., 2022).

### Temperature stress

Environmental temperature is rising due to greenhouse-gas emissions in the atmosphere, where the average global temperature has continuously increased and is predicted to rise by 2°C until 2100 (Raftery et al., 2017; Malhi et al., 2021), causing substantial agricultural production losses worldwide. The major proportion of greenhouse gases has several effects on crops (Mukhtar et al., 2020). A major concentration of CO_2_ is related to increased photosynthesis (i.e., higher growth and plant productivity), however, an increased temperature counteracts this effect by increasing evapotranspiration and crop respiration rate, promoting pest infestation and weed flora, reducing crop duration, and damaging the microbial population and enzymatic activities of the soil (Malhi et al., 2021; Battaglia et al., 2022). Plants induce morphological and biochemical changes to survive to heat stress that leads to oxidative stress caused by an overproduction of ROS, severely affecting growth, development, and yield (Hasanuzzaman et al., 2013). During this type of abiotic stress, plants induce signaling processes to produce osmolytes that sustain cell turgidity by osmotic adjustment and other secondary metabolites to modify the antioxidant system (Arif et al., 2020). High temperatures have detrimental effects on the photosynthetic functions by damaging the oxygen-evolving complex, PSII cofactors, carbon assimilation, and the ATP generation (Allakhverdiev et al., 2008). Additionally, in low-temperature stress, freezing injury and fluidity reduction occurs in cell membranes (Los and Murata, 2004). Positive effects of NPs on plants under temperature stress have been demonstrated by enhancing the photophosphorylation, oxygen evolution, and splitting of water CP43 protein (Pradhan et al., 2013), nitrogen metabolism (Pradhan et al., 2014), photosynthetic capacity (Younis et al., 2020), antioxidant enzymes activity, decreasing lipid peroxidation (Hassan et al., 2018), and restoration of ultrastructural distortions of chloroplasts and the nucleus (Younis et al., 2020).

### Soil contamination with metal traces

The accumulation of toxic HMs and metalloids (e.g., As, Pb, Cd, Cu, Cr, Mn, Hg, Ni, Se, Sb, and Zn) in soil is a severe environmental issue that causes detrimental effects on every living organism (Seleiman and Kheir, 2018; Seleiman et al., 2020). Human activities (e.g., industrial and municipal discharge, mining, smelting, hazardous solid waste disposal, extensive use of agrochemicals) mainly contribute to the accumulation of these compounds (Seleiman et al., 2012; Ali et al., 2019; Shah and Daverey, 2020; Seleiman et al., 2021). Persisting HMs in the soil can absorbed by crops that reaches human consumption, causing cancer (due to DNA damage), vascular disease, dermal disease, gastrointestinal problems, respiratory damage, brain damage, renal disorder, degenerative bone disease, liver damage, depression, mental retardation, cardiovascular and respiratory system disorders, etc. (Ye et al., 2017; Ali et al., 2019; Shah and Daverey, 2020). HMs persist in the soil for many years due to its non-biodegradable nature, and their mobility and availability is controlled by biogeochemical process (i.e., mineralization, precipitation, adsorption, and protonation), the type of soil, and rhizosphere which contains a diverse root microbiome that contributes in soil fertility and is highly affected by these compounds (He et al., 2015; Shah and Daverey, 2020). Several mechanisms have been proposed to counteract the negative effect of this abiotic stress. The immobilization of metal ions by absorption, oxidation, or chemical reduction processes is another aspect to highlight in NPs used for soil remediation in several countries (Shen et al., 2019; Qian et al., 2020; Usman et al., 2020). In plants, the formation of apoplastic barriers, which control the flow of water, ions and oxygen, can be influenced by NPs, decreasing the amount of HMs in the roots (Yang et al., 2013); metal transport genes can be regulated by specific NPs, enhancing the plant’s extracellular barrier to intercept HM (Zhou et al., 2020); organic acids accumulated in the cell walls of roots and leaves can be chelated with HM to reduce the damage of HM stress to plants; and finally the activation of the oxidation defense system influenced by NPs can also alleviate HM stress (Marslin et al., 2017).

### Light stress

Light from sunlight, and recently from artificial sources, is not only essential for plant growth and development through photosynthesis but also in gene expression, production of natural bioactive compounds, and synchronization of the circadian clock (Aguirre-Becerra et al., 2020; Aguirre-Becerra et al., 2021; Xu et al., 2022). Light intensity, direction, quality (wavelength), and photoperiod are sensed by specialized designed proteins that sense light named photoreceptors, triggering chain reactions that have been studied in terms of photomorphogenesis and primary and secondary metabolites production (Alvarado et al., 2019). Five types of photoreceptors have been identified: phytochromes perceiving red (660–700 nm) and far-red (700–750 nm), cryptochromes, phototropins, and members of the Zeitlupe family perceiving blue (495–400 nm) and UV-A (400–315 nm), and UV Resistance Locus 8 (UVR8) sensing (315–280 nm) (Bantis et al., 2018; Alvarado et al., 2019; Aguirre-Becerra et al., 2021). Insufficient or excess light levels can result in plant stress, producing adverse effects that trigger the defense mechanisms for secondary metabolite production (Akula and Ravishankar, 2011; Müller-Xing et al., 2014). Additionally, the impact of UV radiation on organisms has focused the scientific community’s interest due to the depletion of the stratospheric ozone layer and more UV-B, very harmful for most organism, could reach the Earth biosphere (Bernhard et al., 2020).

Photoactive NPs (e.g., cadmium sulfide, gold nanoclusters, and indium phosphide nanoparticles) have been used to construct functional biological−inorganic hybrid systems for improving light absorption, typically transferring light into photogenerated electrons for regulating CO2 fixation and NADPH generation (Li et al., 2020). Moreover, luminescent NPs (e.g., carbon dots, silicon nanoparticles, and nanophosphors) have been involved in plant research to improve photosynthesis efficiency due to their tunable photoluminescence, biocompatibility, low cytotoxicity, and chemical/physical stability (Dong et al., 2019). However, higher concentrations of NPs can show adverse effects, damage the photosynthetic apparatus, inhibit photosynthetic electron transport or CO2 reduction by suppressing Rubisco activity and production of harmful ROS (Jampílek and Kráľová, 2019); therefore, correct doses for achieving enhanced photosynthesis are still under research. Additionally, most experiments evaluate the effect of NPs on photosynthesis due to stress (e.g., temperature, drought, trace metals) conditions or to enhance this biological process under normal light conditions. This measurement is related to the efficiency of light energy conversion; however, just a few experiments were found where light is the stress condition (i.e., low or high light intensities, harmful wavelengths, or not adequate photoperiods). For example, hydroponic wheat *(Triticum aestivum*) seedlings were exposed to UV-B and treated with SiNPs. These compounds alleviated the toxic effects as fresh mass, leaf area, leaf area, leaf fresh and dry mass increased, and lipid peroxidation (as MDA) decreased. UV-B significantly reduced the total chlorophyll, and SiNPs restricted such reductions. Additionally, the intensity and numbers of formazan spots were reduced in the leaves, suggesting a protective role against UV-B-induced generation of H_2_O_2_ (i.e., oxidative stress). Furthermore, internal injuries of leaves related to the distorted palisade and mesophyll layers were reduced; therefore, the reduction in plant leaf thickness was alleviated (Tripathi et al., 2017). Phytotoxicity considerations

The type, size, morphology, crystallinity, concentration, electric-magnetic-optical properties, type of application (i.e., foliar or substrate), and vegetal species determine the phytotoxicity of NPSs. The accumulation of these compounds can lead to an imbalance in the interaction of soil, plant, and microbial communities resulting in phytotoxicity and plant death. NPs mainly accumulate in the soil due to atmospheric deposition, rain erosion, agricultural application, and surface runoff, facilitating their transfer into the plant and moving to different organs (Agrawal et al., 2022). Due to the characteristics of NPs (i.e., size, morphology, and surface charge), they can easily penetrate the vegetal tissues and the human body with possible adverse effects (Ndaba et al., 2022). Risks associated with NPs should be assessed along with their benefits. Most of the mentioned studies evaluated the advantages of using these compounds, but only a few consider the adverse effects and negative downstream implications.

**Figure 2:**
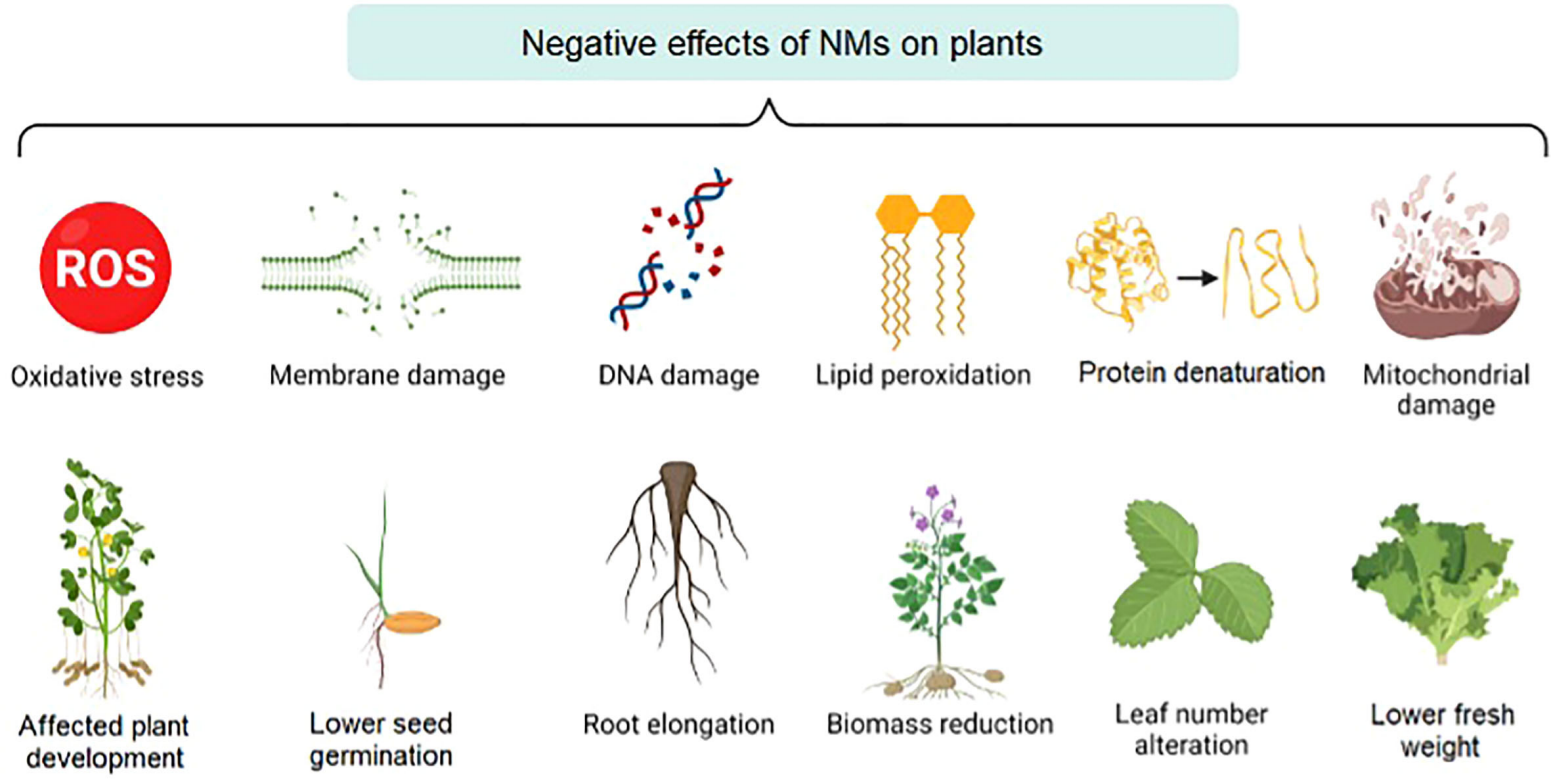
Physiological response of plants by the toxicity mechanism of NPs.

Once NPs move into the plant, mainly by absorption, different chemical elements, and heavy metals could be dissolved, released, and even bio-transformed, generating potentially toxic compounds for the environment (Li and Yan, 2020). As previously discussed, nanotechnology applications in agriculture are potentially beneficial; nevertheless, the diverse nature and characteristics of NPs generate a challenging and not completely understood interaction with living organisms, which can potentially damage the food chain. Additionally, NPs can harm plants by clogging pores and blocking the apoplastic movement causing decreased nutrient absorption, photosynthesis, and hydraulic transfer, DNA damage, and ROS production (Ganie et al., 2021). Plants exposed to NPs have also presented altered physiology (e.g., root and shoot elongation), germination percentage, leaf number, malfunction of cell organelles, tissue damage, structural modification of enzymes, protein denaturation, destruction of cell membranes, and non-beneficial responses to biotic and abiotic stresses (Agrawal et al., 2022). These effects are schematically presented in **Figure 2**.

Knowing how NPs affect plants, humans, and the environment helps generate safe protocols to reduce their adverse effects and create “safer” NPs through the green or biosynthesis approach. Further studies on genetic alterations are needed to clarify toxic effects and the plant-triggered adaptation mechanisms to propose specific doses according to the particular plant and NP to potentiate the beneficial responses (eustress) in the plant physiology and defense mechanisms (secondary metabolism) (Aguirre-Becerra et al., 2021; Ranjan et al., 2021).

## Conclusion and future perspectives

In this manuscript, a general overview was performed on the effect of nanomaterials on the plant resistance to abiotic stress. The implementation of NMs and NPs is one of the main proposed strategies for substituting conventional fertilizers, herbicides, and pesticides in agriculture and enhancing the effect of growth regulators and the defense system of plants. This relatively new technology generates diverse effects depending on the crop, NM, concentration, and type and frequency of application. The particular effect of each NP depends on the size, surface area, thermal stability, crystallinity, concentration, electric-magnetic-optical properties, and origin, where the biosynthesis through the implementation of bacteria, fungi, and plant extracts has resulted in greater effectiveness in plant resistance to abiotic stress and lower phytotoxicity. Additionally, the nutrient diversity, controlled release, and rapid absorption make NPs a promising tool for the agri-food sector, generating enhanced growth and crop yield and accelerating the germination processes.

Different studies indicate that the interaction NP-plant induces reactions that help the plant cope with adverse environmental factors. Among these reactions, higher plant plasticity, generation of secondary metabolites, enhancement of the immune system of plants, and acceleration of plant growth and development, make plants more resilient. Most reactions are favorable for the adaptability of crops to stress situations; however, some reactions are not desirable, and there is little information about them. How NPs and NMs could affect not only the crops but also the environment: substrates, water resources, substrate microorganisms, fauna, flora, and the short, medium, and long-term effect of the consumption of these crops on humans.

Another aspect that has not been completely understood or about which there is little information is the overuse that can lead to the accumulation of NPs in the natural environment. Most of the available information focuses on NPs applied under laboratory and controlled and supervised conditions, with particular and correct disposal of experimental waste material. Therefore, the interaction between NPs and open-air plants and the effect of their accumulation in natural environments is still unknown. As mentioned in this chapter, many variables must be considered when applying NPs to enhance plant resistance to abiotic stress. Many studies and research areas are still to be more involved (e.g., genetics, omics sciences) to achieve secure and effective protocols for using NPs and NMs in agriculture.

## Author contributions

HA-B, KE, CP-G, and AF-P: data acquisition, drafting of the manuscript, and conceptualization; MV-H and AM-A: bibliographic review; AF-P, KE, and CP-G: obtained funds and responsibility for managing and coordinating the planning and execution of the research activity and revision of the manuscript. All authors assisted in the critical review of this manuscript approved the version presented for publication and agreed to be responsible for all aspects of the article.

## Acknowledgments

The authors appreciate the support provided by FONDEC-UAQ 2021 FIN202115 and Autonomous University of Queretaro for the realization of this project.

## Conflict of interest

The authors declare that the research was conducted in the absence of any commercial or financial relationships that could be construed as a potential conflict of interest.

## Publisher’s note

All claims expressed in this article are solely those of the authors and do not necessarily represent those of their affiliated organizations, or those of the publisher, the editors and the reviewers. Any product that may be evaluated in this article, or claim that may be made by its manufacturer, is not guaranteed or endorsed by the publisher.
